# Supplementary material for: Neuromodulation of Astrocytic K+ Clearance
Source: Int J Mol Sci. 2021 Mar 3;22(5):2520. doi: 10.3390/ijms22052520 (PMC7959145; doi:10.3390/ijms22052520)
Supplement: Supplementary file 1 [file ijms-22-02520-s001.pdf]

International Journal of *Molecular Sciences*

MDPI

Supporting Information

Neuromodulation of astrocytic K+ clearance

Alba Bellot-Saez[1], Rebecca Stevenson[1], Orsolya Kékesi[1], Evgeniia Samokhina[1], Yuval Ben-Abu[3], John W. Morley[1] and Yossi Buskila[1,2*]

[1] School of Medicine, Western Sydney University, Campbelltown, NSW 2560, Australia.
[2] International Centre for Neuromorphic Systems, The MARCS Institute, Western Sydney University, Penrith, NSW 2751, Australia.
[3] Projects and Physics Section, Sapir Academic College, D.N. Hof Ashkelon 79165, Israel.

* Correspondence: y.buskila@westernsydney.edu.au; Tel.: +61246203853

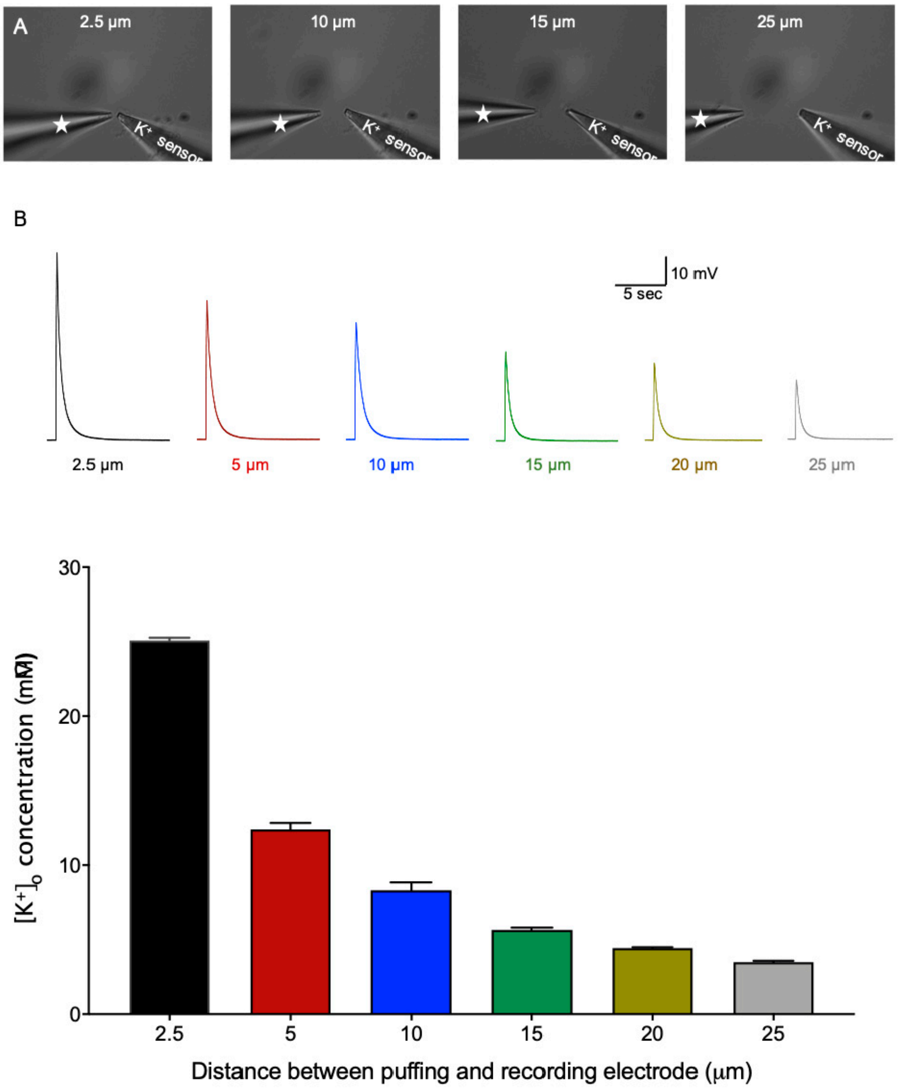

Supplementary Figure S1. The impact of distance between the puffing and recording electrodes on the recorded concentration of $[K^+]_o$. A) sample pictures of the puffing and recording electrodes at different distances. B) sample traces depicting the change in the voltage amplitude following application of 30 mM KCl at different distances. C) Bar graph summarizing the impact of the distance between the recording and puffing electrodes on the overall measured $[K^+]_o$ concentration. Data presented as Mean ± S.E.M.

Table S1. The impact of 5-HT on the K^+ clearance rate. Average values of the $[K^+]_o$ clearance rate (90-10%), amplitude, rise time (10-90%) and top peak area (10%) at all concentrations tested, before and after the application of 5-HT or 5-HT + TTX. Data is reported as mean ± S.E.M.

$[K^+]_o$	Condition	Clearance rate (mM/sec)	Amplitude (mM)	Rise time (sec)	Peak area (mMxsec)
**30 mM**	aCSF	2.04±0.20	6.40±0.53	0.32±0.02	1.25±0.08
**15 mM**	aCSF	0.84±0.06	2.76±0.16	0.34±0.01	0.66±0.04
**5 mM**	aCSF	0.37±0.03	1.07±0.21	0.28±0.03	0.25±0.02
**30 mM**	5-HT	1.33±0.14	6.64 ±0.74	0.34±0.02	1.43±0.08
**15 mM**	5-HT	0.82±0.05	2.73±0.22	0.34±0.02	0.63±0.05
**5 mM**	5-HT	0.34±0.04	0.98±0.03	0.27±0.03	0.26±0.04
**30 mM**	5-HT+TTX	1.29±0.11	6.63±0.61	0.33±0.02	1.40±0.06
**15 mM**	5-HT+TTX	0.79±0.05	2.69±0.19	0.33±0.02	0.67±0.04
**5 mM**	5-HT+TTX	0.39±0.04	0.99±0.05	0.27±0.04	0.25±0.03

Table S2. The impact of DA on the K^+ clearance rate. Average values of the $[K^+]_o$ clearance rate (90-10%), amplitude, rise time (10-90%) and top peak area (10%) at all concentrations tested, before and after the application of DA or DA + TTX. Data is reported as mean ± S.E.M.

$[K^+]_o$	Condition	Clearance rate (mM/sec)	Amplitude (mM)	Rise time (sec)	Peak area (mMxsec)
30 mM	aCSF	2.46±0.28	7.51±1.05	0.27±0.03	1.96±0.15
15 mM	aCSF	1.60±0.25	4.24±0.43	0.27±0.02	0.99±0.08
5 mM	aCSF	0.80±0.11	1.46±0.16	0.26±0.02	0.35±0.02
30 mM	5-HT	1.61±0.26	7.32±0.83	0.28±0.03	2.41±0.17
15 mM	5-HT	1.35±0.17	4.13±0.40	0.29±0.02	1.21±0.11
5 mM	5-HT	0.60±0.09	1.49±0.19	0.27±0.02	0.46±0.04
30 mM	DA+TTX	1.68±0.25	7.25±0.82	0.28±0.02	2.36±0.22
15 mM	DA+TTX	1.21±0.15	4.26±0.41	0.28±0.01	1.10±0.09
5 mM	DA+TTX	0.56±0.08	1.49±0.16	0.27±0.01	0.42±0.04

Table S3. The impact of NA on the K^+ clearance rate. Average values of the $[K^+]_o$ clearance rate (90-10%), amplitude, rise time (10-90%) and top peak area (10%) at all concentrations tested, before and after the application of NA or NA + TTX. Data is reported as mean ± S.E.M.

$[K^+]_o$	Condition	Clearance rate (mM/sec)	Amplitude (mM)	Rise time (sec)	Peak area (mMxsec)
30 mM	aCSF	1.42±0.14	5.99±0.26	0.40±0.02	1.20±0.08
15 mM	aCSF	0.87±0.05	2.56±0.15	0.35±0.02	0.54±0.06
5 mM	aCSF	0.44±0.04	0.92±0.06	0.30±0.02	0.25±0.04
30 mM	5-HT	0.80±0.06	5.94±0.45	0.39±0.03	1.45±0.07
15 mM	5-HT	0.70±0.06	2.52±0.11	0.36±0.02	0.78±0.08
5 mM	5-HT	0.42±0.04	0.92±0.05	0.29±0.02	0.30±0.04
30 mM	NA+TTX	0.90±0.07	5.76±0.41	0.38±0.03	1.54±0.10
15 mM	NA+TTX	0.65±0.05	2.49±0.09	0.35±0.02	0.77±0.08
5 mM	NA+TTX	0.42±0.04	0.91±0.04	0.30±0.03	0.28±0.05

Table S4. The impact of Histamine on the K^+ clearance rate. Average values of the $[K^+]_o$ clearance rate (90-10%), amplitude, rise time (10-90%) and top peak area (10%) at all concentrations tested, before and after the application of Histamine or Histamine+TTX. Data is reported as mean ± S.E.M.

$[K^+]_o$	Condition	Clearance rate (mM/sec)	Amplitude (mM)	Rise time (sec)	Peak area (mMxsec)
30 mM	aCSF	2.02±0.38	6.52±0.77	0.31±0.01	0.74±0.03
15 mM	aCSF	1.12±0.09	3.50±0.32	0.28±0.01	0.51±0.04
5 mM	aCSF	0.51±0.05	0.93±0.09	0.26±0.01	0.15±0.01
30 mM	Histamine	1.15±0.14	6.63±0.57	0.32±0.01	1.04±0.06
15 mM	Histamine	0.84±0.08	3.49±0.56	0.27±0.01	0.72±0.04
5 mM	Histamine	0.30±0.02	0.93±0.14	0.25±0.02	0.19±0.01
30 mM	Histamine +TTX	1.19±0.16	6.49±0.61	0.32±0.01	1.09±0.04
15 mM	Histamine +TTX	1.09±0.12	3.41±0.54	0.27±0.02	0.63±0.05
5 mM	Histamine +TTX	0.46±0.03	0.91±0.07	0.26±0.01	0.17±0.01

Table S5. The impact of Acetylcholine on the K^+ clearance rate. Average values of the $[K^+]_o$ clearance rate (90-10%), amplitude, rise time (10-90%) and top peak area (10%) at all concentrations tested, before and after the application of ACh or ACh + TTX. Data is reported as mean ± S.E.M.

$[K^+]_o$	Condition	Clearance rate (mM/sec)	Amplitude (mM)	Rise time (sec)	Peak area (mMxsec)
30 mM	aCSF	2.02±0.38	6.52±0.77	0.31±0.01	0.74±0.03
15 mM	aCSF	1.12±0.09	3.50±0.32	0.28±0.01	0.51±0.04
5 mM	aCSF	0.51±0.05	0.93±0.09	0.26±0.01	0.15±0.01
30 mM	ACh	1.15±0.14	6.63±0.57	0.32±0.01	1.04±0.06
15 mM	ACh	0.84±0.08	3.49±0.56	0.27±0.01	0.72±0.04
5 mM	ACh	0.30±0.02	0.93±0.14	0.25±0.02	0.19±0.01
30 mM	ACh +TTX	1.19±0.16	6.49±0.61	0.32±0.01	1.09±0.04
15 mM	ACh +TTX	1.09±0.12	3.41±0.54	0.27±0.02	0.63±0.05
5 mM	ACh +TTX	0.46±0.03	0.91±0.07	0.26±0.01	0.17±0.01
